# Neurodegenerative Microbially-Shaped Diseases: Oxidative Stress Meets Neuroinflammation

**DOI:** 10.3390/antiox11112141

**Published:** 2022-10-28

**Authors:** Diana Filipa Silva, Nuno Empadinhas, Sandra Morais Cardoso, Ana Raquel Esteves

**Affiliations:** 1 CNC—Center for Neuroscience and Cell Biology and CIBB—Center for Innovative Biomedicine and Biotechnology, University of Coimbra, 3004-504 Coimbra, Portugal; 2 IIIUC—Institute for Interdisciplinary Research, University of Coimbra, 3030-789 Coimbra, Portugal; 3 Institute of Cellular and Molecular Biology, Faculty of Medicine, University of Coimbra, 3004-504 Coimbra, Portugal

**Keywords:** Alzheimer’s disease, Parkinson’s disease, inflammation, oxidative stress, microbiome, gut–brain axis

## Abstract

Inflammation and oxidative stress characterize a number of chronic conditions including neurodegenerative diseases and aging. Inflammation is a key component of the innate immune response in Alzheimer’s disease and Parkinson’s disease of which oxidative stress is an important hallmark. Immune dysregulation and mitochondrial dysfunction with concomitant reactive oxygen species accumulation have also been implicated in both diseases, both systemically and within the Central Nervous System. Mitochondria are a centrally positioned signalling hub for inflammatory responses and inflammatory cells can release reactive species at the site of inflammation often leading to exaggerated oxidative stress. A growing body of evidence suggests that disruption of normal gut microbiota composition may induce increased permeability of the gut barrier leading to chronic systemic inflammation, which may, in turn, impair the blood–brain barrier function and promote neuroinflammation and neurodegeneration. The gastrointestinal tract is constantly exposed to myriad exogenous substances and microbial pathogens, which are abundant sources of reactive oxygen species, oxidative damage and pro-inflammatory events. Several studies have demonstrated that microbial infections may also affect the balance in gut microbiota composition (involving oxidant and inflammatory processes by the host and indigenous microbiota) and influence downstream Alzheimer’s disease and Parkinson’s disease pathogenesis, in which blood–brain barrier damage ultimately occurs. Therefore, the oxidant/inflammatory insults triggered by a disrupted gut microbiota and chronic dysbiosis often lead to compromised gut barrier function, allowing inflammation to “escape” as well as uncontrolled immune responses that may ultimately disrupt mitochondrial function upwards the brain. Future therapeutic strategies should be designed to “restrain” gut inflammation, a goal that could ideally be attained by microbiota modulation strategies, in alternative to classic anti-inflammatory agents with unpredictable effects on the microbiota architecture itself.

## 1. Introduction

**Figure 1:**
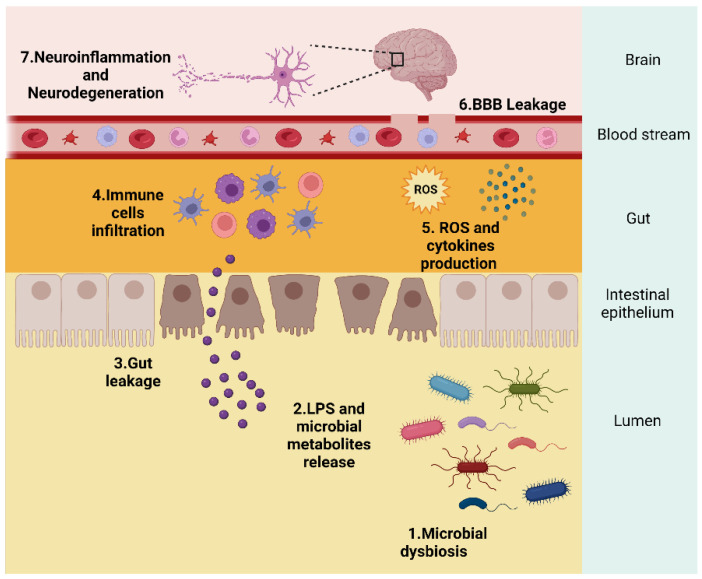
Signalling mechanisms between gut microbiota and the brain. Gut microbiota dysbiosis (1) results in increased LPS and microbial metabolites in the lumen (2) that are released from bacteria which damages intestinal epithelial cells culminating in gut leakage (3). This increase in the gut barrier permeability prompts immune cells infiltration (4) that can also release ROS leading to exaggerated oxidative stress and liberate cytokines that circulate from the blood to the brain (5). This systemic inflammation and ROS overproduction may in turn impair the blood–brain barrier (6) and promote neuroinflammation culminating in neurodegeneration (7). Created by Biorender.

**Figure 2:**
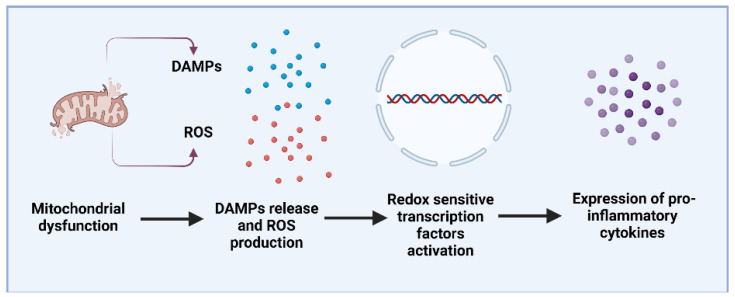
Mitochondria has a signalling hub for inflammatory responses. Mitochondrial dysfunction leads to an overproduction of ROS and release of DAMPs that can activate redox sensitive transcription factors such as transcription factor nuclear factor κB (NF-κB) and enhance the expression of inflammatory genes leading to the increase in pro-inflammatory cytokines and mediators. Created by Biorender.

A great body of evidence has unequivocally linked oxidative stress and chronic inflammation to the pathogenesis of neurodegenerative disorders such as Parkinson’s disease (PD) and Alzheimer’s disease (AD) [1,2]. Recently, the microbiota–gut–brain axis was added to the well-known processes mediating the neurodegenerative process in these diseases [3,4]. Indeed, significantly higher levels of lipopolysaccharides (LPS) were detected in the plasma of patients with neurodegenerative diseases when compared with age-matched controls, with a correlation between LPS and NADPH oxidase 2 (NOX2) activation [5]. Gut microbiota dysbiosis results in increased LPS in the periphery, leads to NOX2 activation and reactive oxygen species (ROS) overproduction, favouring the progression of neurodegenerative disorders [5] (Figure 1). A healthy microbiota regulates intestinal health and homeostasis but when this ecosystem is disturbed, pathogens and/or pathobionts may release LPS and enterotoxins, damaging intestinal epithelial cells and leading to the activation of inflammatory responses which will ultimately damage the gut epithelial barrier [6]. Inflammatory cells at the local of damage liberate an ROS leading to exaggerated oxidative stress. On the other hand, ROS and reactive nitrogen species (RNS) can initiate intracellular signalling cascade that enhances proinflammatory gene expression [7]. Thus, inflammation and oxidative stress are tightly linked to the neurodegenerative process. Extensive data showed the simultaneous existence of low-grade chronic inflammation and oxidative stress in chronic diseases including diabetes, cardiovascular and neurodegenerative diseases [8]. Moreover, mitochondrial dysfunction is a central feature in the pathophysiology of neurodegenerative disorders [9]. Dysfunctional mitochondria activate inflammation through the release of damage-associated molecular patterns (DAMPs), triggering innate immune responses in both resident and infiltrating cells. The release of DAMPs leads to the activation of NOD-like receptors (NLRs) and Toll-like receptors (TLRs), promoting inflammatory cytokine, chemokines, and reactive oxygen species production, impacting disease progression [10] (Figure 2).

The present review will explore the basic aspects of oxidative stress and inflammation and their interrelationship in a context of PD and AD disorders. Moreover, we will discuss data regarding Enteric Nervous System (ENS) and Central Nervous System (CNS) oxidative stress, and inflammation alterations that might be responsible for aggravating PD and AD pathology, and the mediator(s) of the transmission of the pathology from the gut to the brain. Ultimately, we aim to gather insights of how the modulation of gut microbiota can influence gut dysbiosis, with a focus on neurodegenerative diseases.

## 2. Oxidative Stress and Chronic Inflammation Interdependence in Neurodegenerative Disorders

### 2.1. Alzheimer’s Disease

#### 2.1.1. Pathogenesis of Alzheimer’s Disease

Sporadic AD (sAD) is a neurodegenerative disorder whose pathophysiology is still a matter of intense research, with no disease-modifying treatment available [11]. It is now recognized that the aetiology of sAD is multifactorial, with interaction between genetic and environmental factors [12]. AD neuropathology is defined by the deposition of extracellular amyloid-beta (Aβ) plaques and intracellular neurofibrillary tangles (NFT) of Tau [13]. Nevertheless, the relationship between Aβ and neurodegeneration is weak with the appearance of neurodegeneration in the absence of Aβ and, in opposition, the presence of substantial content of Aβ in elderly individuals with no dementia. Moreover, clinical trials that had successfully reduced Aβ levels in the brain failed to slow cognitive decline, even so that some had adverse effects with increased infection rates [14]. These observations lead to the hypothesis that Aβ might be an anti-microbial peptide (AMP), an innate immune trait that consists in the fibrillation of Aβ to create a protective barrier against infectious agents [14] such as bacteria and viruses. In vitro studies showed that Aβ is a potent AMP against a number of clinically relevant microorganisms and, remarkably, AD temporal lobe homogenates had significantly higher anti-microbial activity in comparison with aged-matched controls [15]. Interestingly, the antimicrobial power of Aβ even exceeded LL-37 against certain microorganisms [15], a known AMP with strong antimicrobial activity both in vitro and in vivo [16,17,18]. Thus, 5XFAD transgenic mice (overexpresses human amyloid precursor protein (APP) with three familial AD mutations and human presenilin 1 (PS1) with two familial AD mutations) that recapitulate many AD-related phenotypes, exposed to *Salmonella Typhimurium* have higher survival rates than mice lacking APP [19]. The authors highlight that Aβ oligomerization and fibrillization mediate Aβ’s antimicrobial activity, since cerebral infection of the 5XFAD mice resulted in rapid seeding and accelerated Aβ deposition, which colocalizes with *Salmonella Typhimurium*. Although Aβ deposition might represent the first protective shield against AD, it comes with a high cost. Extracellular Aβ deposition is a potent inflammatory stimulus, by binding to pattern recognition receptors (PRR) on the surface of microglia membrane, it triggers innate immunity activation [20]. TLRs, part of PRR family, mediate phagocytosis of Aβ by microglia. TLRs recognize PAMPs (pathogen-associated molecular patterns) and DAMPs, activating a cascade of events that activate NF-κB, leading to the production and release of inflammatory cytokines. Chronic activation of innate immunity in AD, causes malfunctioning and culminates in neuronal death [20]. Among the TLR family, expression levels of TLR2 have been found to be increased in the brains of both AD patients and animal models [21], suggesting an important impact on AD progression. TLR2 is activated by axonal injury and oligomeric and fibrillar Aβ, causing M1 microglial activation, thus resulting in secretion of proinflammatory cytokines and neuronal damage [22]. Blocking TLR2 function with an antibody was reported to neutralize Aβ-induced pro-inflammatory cytokine release and thus, neurodegeneration [23].

Regarding Tau pathology contribution for AD progression, Tau belongs to the family of microtubule-associated proteins (MAPs) and is highly expressed by neurons with preferential axonal localization, associated with microtubules for axonal transport [24]. The presence and extent of hyperphosphorylated tau NFT pathology is correlated with disease duration and severity of cognitive symptoms [25]. In vitro studies showed Tau promotes tubulin polymerization, influencing microtubule dynamics [26], thus, its detachment from microtubules during AD progression, affects axonal transport and renders Tau accessible for phosphorylation by kinases [27]. Widespread of Tau has been shown in various in vitro and in vivo models by a self-replicating mechanism, first described in prion diseases, where pre-existing aggregates (seeds) can imprint their pathological conformation on naïve proteins that spread to neighbouring cells [28]. Data supporting this hypothesis have demonstrated that tau aggregates, isolated from a variety of different human tauopathies, have been successfully transmitted to transgenic mouse and cell models, suggesting that they adopt a prion conformation upon misfolding [29,30,31]. These soluble Tau oligomers co-localize with activated microglial cells and reactive astrocytes, observed in both mouse models of Tauopathy and AD/frontotemporal lobar dementia (FTLD) patients’ brains [32]. In microglial cultures exposed to misfolded truncated Tau, this is sufficient to induce pro-inflammatory cytokines production, namely interleukin (IL)-6, IL-1β, TNF-α, through NF-κB and MAPK signalling pathways [33]. Evidence from in vivo model emphasized that pathological Tau could promote IL-1β secretion by activating NLRP3 (nucleotide binding oligomerization domain, leucine-rich repeat and pyrin domain-containing protein 3 inflammasome [34].

#### 2.1.2. Oxidative Stress and Inflammaging in Alzheimer’s Disease: Brain and Gut Implications

Adding to the myriad of pathways and players that mediate AD aetiology and progression, current evidence points that alterations in the brain-gut axis, that reflects the bidirectional, constant communication between the CNS and the gastrointestinal tract, contribute to the development of AD [35]. Recent studies indicate that alterations in the gut microbiota composition induce increased permeability of the gut barrier and immune activation leading to systemic inflammation. Chronic inflammation may in turn damage the blood–brain barrier (BBB) and promote neuroinflammation, neural injury, and ultimately neurodegeneration [35,36]. The microbiota–gut–brain axis refers to a cross-talk system between the gut microbiota, the ENS, and the brain [37], involving the autonomic, neuroendocrine and immune systems as well as bacteria metabolites and neuromodulatory molecules [38]. It is now well recognized that oxidative stress is a major hallmark of AD pathology, possibly driven by mitochondrial dysfunction [39]. Markers of oxidative damage, such as lipid peroxidation and protein carbonyls, have been detected in brain samples of both AD animal models [40] and patients [41]. Although a much less explored subject, brain/CNS modulates the levels of oxidative stress within the gut via vagus nerve, namely the cholinergic anti-inflammatory pathway [37]. Vagal stimulation causes the release of acetylcholine (ACh) in the distal end of the vagal efferents, downregulates pro-inflammatory cytokines release by macrophages [42], thus, possibly reducing oxidative stress within the gut. In AD, several neurotransmitter systems are defective, including serotonergic, noradrenergic, and, the most severely affected, the cholinergic system [43], therefore representing a mechanism of oxidative stress imbalance, from the brain to the gut. Clinical strategies that target vagus nerve have been put in place, indeed a clinical trial showed that vagus nerve stimulation is well tolerated and had positive effects in the cognition of AD patients, with beneficial effects maintained for a period of 12 months follow-up [44]. Recently, vagus nerve stimulation was applied to amyloid precursor protein/presenilin-1 (APP/PS1) transgenic mice, showing a clear effect on microglia from activated state to a more neuroprotective phenotype [45], constituting a strategy to tune the inflammatory signalling [46]. Given the described effects, one can speculate on the protective effects concerning oxidative damage, since microglia are a major source of ROS.

It is now well accepted that AD pathology is not brain limited. In fact, peripheral markers of oxidative stress are present in plasma and blood cells of AD subjects [47,48,49]. A body of evidence has reported that intestinal dysbiosis and the “leaky gut syndrome” in AD, measured by increased calprotectin in faecal material from AD subjects [50], represents a key route for microbiome-derived molecules to reach the brain. On the other hand, faecal content in short-chain fatty acids (SCFAs), namely propionate and butyrate, are reported to be decreased in AD subjects [51]. Propionate, butyrate and valeric acid showed efficacy in reducing Aβ peptides aggregation, diminishing its potential neurotoxic capacity [52]. Aβ peptides can enter the mitochondria [39] worsening mitochondrial dysfunction and thus, increasing oxidative damage [53] both in peripheral tissues and in the brain, as mitochondria are major source of ROS. As discussed previously, ACh production is impaired in AD. A number of gut bacteria strains have been described as neurotransmitter producers, which are proposed to modulate nervous system activity [54]. In AD subjects, reduced levels of some bacterial taxa such as *Bifidobacterium*, are involved in the production of AD-associated neurotransmitters such as ACh [55] demonstrating a potential link between gut dysbiosis, neurotransmitter dysregulation and oxidative stress that spreads from the periphery to the brain. Other strains such as *Escherichia coli* (*E. coli*), with increased abundance in AD subjects’ faeces, can convert nitrate and nitrite into nitric oxide leading to increased permeability of the BBB. Nitric oxide reacts with superoxide to form peroxynitrite, a potent oxidizing agent that contributes to oxidative stress in AD brains [56].

As previously mentioned, oxidative stress is a major player in AD and is intimately linked to chronic inflammation. Within the brain, AD pathology players are well recognized; nevertheless, the chronological arise of events is not consensual. According to the inflammation hypothesis for AD, inflammation, and specially neuroinflammation, play a pivotal role in progression of neuropathological hallmarks observed in AD [57]. Reports of immune-related proteins as well as reactive microglia and astrocytes located in close proximity to Aβ plaques, render inflammation a striking event in AD [58]. Aβ has been shown to localize and accumulate in the mitochondria [59,60], resulting in increased mitochondrial toxicity, namely by the deregulation of mitochondrial respiration and consequent ROS overproduction. Within the mitochondria, Aβ peptides interact with dynamin-1-like protein (Drp1), causing excessive mitochondrial fragmentation [61]. Mitochondrial dysfunction-derived oxidative damage starts acting on mitochondria itself, affecting molecules such as mitochondrial DNA (mtDNA) and cardiolipin. Oxidative damage of mtDNA has long been shown in AD [62], causing mtDNA fragments to be released from the mitochondria into the cytosol, upon opening of the permeability transition pore [63]. Regarding cardiolipin it was documented that in brains of AD patients there is increased uptake of translocator protein (TSPO) ligand [11C]vinpocetine, in diseased regions [64]. TSPO oxidizes cardiolipin through ROS generation [65] and is released into the cytosol and extracellular milieu. In line with these observations, recent hypothesis state that mitochondrial constituents, such as mtDNA and cardiolipin, might function DAMPs which could trigger innate immune responses during pathological processes of AD [66]. Indeed, as mitochondria are considered to have originated from α-proteobacteria through endosymbiosis [67], many similarities exist between mitochondrial and bacterial constituents. DAMPs activate TLRs and their coreceptors, creating an oxidative and neuroinflammatory environment in AD via excessive production and release of proinflammatory cytokines, ROS, and NO [68]. Post mortem brains of AD patients show high levels of inflammatory mediators, such as proinflammatory cytokines and chemokines: IL-1, IL-6, TNF-α, MIP-1β, complement activation products, and oxygen radicals [69]. Microglia respond to DAMPs by producing of ROS, which becomes chronically elevated in AD [70]. Microglia also phagocytize Aβ and become pro-inflammatory in the process, expressing cytokines including IL-1β, IL-6, and TNF-α. Similarly, activated microglia are spatially correlated with Tau pathology and are capable of recognizing and clearing aggregated tau [71]. Further, activated microglia promote tau phosphorylation and aggregation by activation of the NLRP3 inflammasome and downstream activation of kinases such as GSK-3β, in response to Aβ overproduction [72]. Interestingly, after a stroke episode, there is the release of DAMPs and cytokines from activated microglia near the ischemic brain tissue. This process and the activation of the vagus nerve will induce gut dysmotility, gut dysbiosis and increased gut permeability [73], proving that neuroinflammation has an impact on gut health.

Regarding the inflammation response driven by Aβ in the gut, contribution to the progression of AD, much work is still to be carried out. Nevertheless, there are observations that point to Aβ playing a role on regulating cholesterol and lipoproteins in the gut. Aβ peptides and Aβ precursor protein were found exclusively in absorptive epithelial cells of the small intestine [74]. Moreover, Caco-2 cells express APP, secrete Aβ 1–40 and Aβ 1–42 upon LPS stimulation and respond by secreting IL-6 cytokine [75]. In vivo experiments showed that Aβ is secreted by enterocytes as an apolipoprotein component of chylomicrons which are large triglyceride-rich lipoproteins produced in enterocytes from dietary lipids [76]. Most important, it was demonstrated that Aβ oligomers, injected into the gut of mice, were internalized by enteric neurons inducing alterations in gastric function. After 1 year the injected Aβ oligomers were present in the vagus nerve and in the brain, and mice exhibited gastrointestinal (GI) dysfunction and cognitive deficits, opening the possibility to Aβ oligomers being produced primarily at the GI tract, years ahead of the CNS manifestation of AD [77]. Another study showed that Aβ, Tau fibrils and AD patient brain lysates injected into the gut of 3 × Tg AD mice, cause Aβ and tau pathology to spread from the colon into the brain through the vagus nerve, initiates an inflammatory response that activates C/EBPβ/δ-secretase, resulting in AD pathology with cognitive impairment [78]. Interestingly, the abundance of certain taxa of gut bacteria are associated with disease progression. Data from streptozotocin-induced diabetic rats showed that orally administration of *Akkermansia muciniphila*, an intestinal commensal bacterium known to maintain a healthy gut barrier function and provide several beneficial effects to the hist physiology, also improves liver function, alleviates oxidative stress, namely through the measurement of malondialdehyde levels, leading to a suppression in the inflammation, ameliorating type 2 diabetes [79], a known risk-factor for AD. The administration of *A. muciniphila* was shown to suppress the overall gut infiltration of mononuclear leukocytes and reduce the levels of TLR2, TLR4 [80], and proinflammatory cytokines, including TNF-α, IL-1α and IL-6 [81]. Although without consensus, *A. muciniphila* was shown to be altered in the gut of AD mouse models [82,83]. APP/PS1 mice treated with *A. muciniphila* displayed reduction in Aβ 40–42 levels in the cerebral cortex and cognitive improvements [84]. These observations led to the hypothesis that gut dysbiosis leads to systemic and neuroinflammation, and subsequently, contributing to cognitive decline [85] in AD and other neurodegenerative disorders.

#### 2.1.3. Is Microbial Infection a Risk Factor for Alzheimer’s Disease?

Alterations in the composition of gut microbiota have been associated with the development of several metabolic diseases including diabetes, obesity [86], and also neurological disorders such as autism spectrum disorder [87] and PD [88]. With respect to AD, data available are quite diverse. A study performed in cognitively impaired patients with brain amyloidosis, demonstrated that the abundance of the pro-inflammatory taxon, *Escherichia*/*Shigella*, and a reduction in the abundance of the anti-inflammatory taxon, *E. rectale*, is likely to be involved with the increase in peripheral inflammatory state and brain Aβ deposition [89]. Another study performed 16S ribosomal RNA (rRNA) gene sequencing of DNA collected from faecal samples of subjects with and without AD diagnosis, and correlated these results with cerebrospinal fluid (CSF) biomarkers of AD. Here the investigators showed that the phylum *Firmicutes* and *Bifidobacterium* genus is decreased in the AD group whereas phylum *Bacteroidetes* has an increased relative abundance, which correlates with the ratio of phosphorylated Tau/Aβ42 [90]. More recently, another report found that AD patients showed a decrease in *Bacteroides*, *Lachnospira* and, an increase in the pro-inflammatory bacteria *Prevotella* [91]. Concerning AD animal models, similar results were obtained regarding bacteria abundance, with APP/PS1 mice, which display higher abundance of *Escherichia-Shigella*, suggesting higher systemic inflammation [92]. Further, another study using 8–12-month-old APPswe/PS1E9 mice point to an increase in the relative abundance of *Verrucomicrobia* and *Proteobacteria* whereas *Ruminococcus* and *Butyricicoccus* compared to age-matched control mice [93]. Regarding 5 × FAD mice, an increased *Firmicutes*/*Bacteroidetes* ratio was evident at the age of nine weeks [94]. The described changes across various models of AD are correlated with SCFAs abundance or have been observed in patients with metabolic syndromes, known as risk factors for AD. As discussed in the previous section, exacerbated inflammation within the gut, due to microbiota alterations, provokes intestinal barrier dysfunction with leakage of inflammatory components into the periphery, as well as microbial translocation, possibly contributing to AD progression. Intriguingly, in the last decade, a body of evidence has been proposing that oral infection, namely periodontitis, contributes to cognition decline [95]. Periodontitis is a common chronic microbial infection in humans, involving various bacteria, and is characterized by the loss of supporting tissue due to local inflammation and, consequently, causes loss of teeth [95]. Oral microbes have been pointed as potential players in AD pathology progression [96]. *Porphyromonas gingivalis* (*P. gingivalis*) was found in post mortem AD brains and authors discuss on the premise that bacteria translocation from the oral cavity into the brain will activate microglia and start a pro-inflammatory cascade with the release of cytokines, ROS among others, exacerbating the ongoing disease-related inflammation [96]. During periodontitis, pro-inflammatory molecules mobilize neutrophils/macrophages from blood vessels for migration to the area of bacterial invasion [97]. There is a physical connection between the oral and nasal cavities, extending onto the superior nasal conchae and nasal septum and contains neurosensory cells and olfactory glands. The cribiform plate of the ethmoid bone is the porous barrier between the nasal passages and the brain itself, behind which is located the anterior part of the enthorhinal cortex area, which leads to the hippocampus [97], both affected during AD progression. Studies using orally infected ApoE-/- mice with *P. gingivalis*, *Treponema denticola* and *Tannerella forsythia* showed that only *P. gingivalis* was found in the brain of these mice, which highlights that specific oral pathogens can alter normal functioning of the brain, contributing to build the inflammatory response and, consequently, neuronal injury [98]. Most important, a longitudinal study evaluated serum antibody levels of bacteria of periodontal disease in individuals that did not have a diagnosis of dementia at the baseline. The authors reported that antibody levels of *Fusobacterium nucleatum* and *Prevotella intermedia* were significantly increased at baseline serum collection, before the diagnosis of neurological disease [99], highlighting that periodontal disease could potentially contribute to the risk of AD onset or contribute to its progression. Thus, the physical connection between the nasal and oral cavities along with disrupted BBB during aging and dementia [100] can potentially facilitate the passage of periodontitis microbes from the systemic circulation into the brain. Periodontitis contribution to AD progression may be linked to in vitro evidence pointing to the fact that *P. gingivalis* express proteolytically active proteases that enable cleavage of the Aβ precursor and Tau resulting in the formation of amyloid-β and neurofibrillary tangles [101]. This observation highlights the likely determinant effect of bacteria within the brain for AD progression. Likewise, *E. coli* was identified in AD brain tissue associated with Aβ pathology [102]; therefore, we can theorize that *P. gingivalis*, that was also found in AD brain samples [103], may also have an impact on Aβ pathology and the downstream AD progression.

### 2.2. Parkinson’s Disease

#### 2.2.1. Pathogenesis of Parkinson’s Disease

PD is a progressive multifactorial neurodegenerative disorder in which the majority of cases are sporadic with unknown aetiology. It is classically associated with motor symptoms that initiate after a loss greater than 80 % dopaminergic (DA) neurons in the substantia nigra (SN) and nigrostriatal pathway dysfunction. It is now known that PD has a very long pro-dromal phase characterized by non-motor symptoms including depression, sleep disturbances mainly rapid eye movement sleep disorder, obstipation, and GI dysfunction [104]. Interestingly, a cohort of newly diagnosed PD patients displayed abnormal intestinal permeability [105]. Moreover, different human cohorts have shown that inflammatory bowel disease might increase the risk of PD [106]. Hence, accumulating evidence demonstrates that the pathogenesis of PD may also relate to intestinal inflammation.

Nevertheless, the prevalent neuropathological features of the PD brain include aggregation of a presynaptic protein, alpha-synuclein (ASYN), that accumulates in intraneuronal inclusions named Lewy Bodies (LBs) in the surviving neurons and progressive degeneration of DA neurons in the SN [107]. Multiple shreds of evidence revealed that LBs are not restricted to the brain. The first report was in the late 1980s and was performed in PD autopsied specimens which generated evidence of ASYN aggregates in the ENS [108]. Since then, an increasing number of studies has showed and characterized the presence of ASYN in the ENS and, as well, evaluated its use as a potential biomarker for PD development. Hypothetically, abnormal ASYN begins to accumulate in the GI tract and is transported to the CNS via the retrograde transport system in the vagus nerve. Remarkably, mounting evidence strongly suggests that PD, in some cases, can initiate through the bidirectional communication between the gut–brain axis [109,110,111]. In 2014, the first experimental evidence that different forms of ASYN can propagate from the gut to the brain via the vagus nerve was provided [112]. Moreover, colonic biopsies from PD patients show increased expression of proinflammatory cytokines and glial markers, which was correlated with the accumulation of ASYN [113]. To corroborate the involvement of the gut–brain axis in PD, a report demonstrated that patients that underwent full truncal vagotomy seem to have a reduced risk for developing PD [114]. In addition, alterations in the gut microbiome composition have been reported in PD patients [115,116]. Overall, more pro-inflammatory gut bacteria, such as LPS-producing *Proteobacteria*, and less anti-inflammatory butyrate-producing gut bacteria are observed in PD patients. Remarkably, severity of some symptoms is associated with alterations in the bacterial composition. For instance, severity of symptoms such as postural instability is associated with alterations in the abundance of certain species of *Enterobacteriaceae* [117]; likewise, severity of motor and nonmotor symptoms is associated with a reduction in *Lachnospiraceae* [118]. More importantly, alterations in the gut microbiota can compromise normal signal transmission between the gut and the brain. In fact, bacteria in the gut can produce many neurotransmitters and neuromodulators such as GABA, serotonin and dopamine that can enter the blood circulation and their precursors can cross the damaged BBB to the brain and participate in the synthesis cycle of neurotransmitters in the brain [119,120]. For instance, histamine concentrations were found to be augmented in the putamen, SN and globus pallidus which was correlated with an increased abundance of histamine producing bacteria in the stools of PD patients [121]. Additionally, an increase in GABA in the basal ganglia and thalamus of PD patients was associated with an increase in bacteria (*Parabacteroides* and *Bacteroides*) that actively express GABA in stool samples from PD patients [122] which was positively correlated with the degree of bradykinesia and rigidity in PD patients [123]. In addition, the gut microbiota can convert substrates into different metabolic products such as SCFAs [124] that can influence the brain indirectly by regulating immune function and inflammation. Interestingly, gut microbiome alterations are associated with alterations in faecal SCFA concentrations and can contribute to the reduced gastrointestinal motility observed in PD patients [124]. Remarkably, PD patients show decreased levels of sodium butyrate which was shown to protect DA neurons from degeneration [125]. SCFAs, including butyrate, can modulate the production of catecholamines by modifying tyrosine hydroxylase gene expression, DA levels and the corresponding animal behaviour [126].

Moreover, gut microbiota imbalance can culminate in chronic inflammation, oxidative stress and apoptosis, all proven aggravators of neurodegenerative disorders like PD. Mitochondrial dysfunction leading to mitochondrial ROS production and mtDNA release have been responsible for subsequent amplification of inflammatory responses [127]. Furthermore, gut dysbiosis leads to chronic low-grade inflammation in the gut, which may ultimately trigger BBB leakage, immune cell activation and inflammation, and ultimately neuroinflammation in the CNS [128]. Remarkably, the increased intestinal permeability in PD results in increased oxidative stress and accumulation of enteric ASYN [105]. Many intestinal diseases are initiated and promoted by oxidative stress, including inflammatory bowel disease, which as mentioned before might increase the risk for PD [129].

#### 2.2.2. Oxidative Stress and Inflammaging in Parkinson’s Disease: Brain and Gut Implications

Over the years, accumulating evidence posits that mitochondria plays a central role in the development of PD pathologic cellular events. In fact, defects in the mitochondrial respiratory chain complex I have been found in post mortem brains from patients with sporadic PD [130].

Interestingly, PD brain biopsies revealed that mitochondrial complex I has oxidatively damaged subunits, which prevents its proper assembly and function [131]. In addition, post mortem samples from the SN of PD subjects show enhanced basal lipid peroxidation and altered glutathione metabolism [132]. A significant reduction in catalase and SOD1 enzymatic function is also observed in the SN of PD patients leading to hydrogen peroxide and superoxide radical anion accumulation [133,134]. As a result, there is a severe and widespread antioxidant system deficit in the SN of PD patients which leads to enhanced ROS production. Environmental toxins such as 1-methyl-4-phenyl-1,2,3,6-tetrahydropyridine (MPTP) and rotenone are known to induce parkinsonism by inhibiting mitochondrial complex I from the respiratory chain, promoting significant ROS generation, reducing ATP synthesis, and culminating in cell death [135]. Moreover, several common PD-related mutations have been implicated in oxidative damage and mitochondrial dysfunction, including DJ-1, PINK-1, LRRK2, PARK2, GBA-1, and ATP13A2 [136,137,138]. Other than an energy source, mitochondria have become a crucial signalling core for managing the signalling networks associated with innate immunity and inflammation. Mitochondria as bacterial endosymbionts maintain some pathogen-associated molecular patterns and release damage-associated molecular patterns triggering basal cytokines and inflammatory mediators release [139]. Neuroinflammatory events in PD were first reported in 1988 where it was observed the presence of activated microglial cells and inflammatory macrophages, as well as proinflammatory cytokines in post mortem brain samples of the SN of PD patients [140]. Later on, Langston and colleagues reported the accumulation of activated microglia around DA neurons in post mortem brains from MPTP-induced parkinsonism humans [141]. Interestingly, the number of MHC class II-positive microglia in the SN increased as the neuronal degeneration proceeded [142]. Not only ROS can initiate intracellular signalling cascade that increases proinflammatory gene expression; but also inflammatory cells can release ROS leading to massive oxidative stress. Hence, activated microglial cells are an important source of ROS and pro-inflammatory cytokines leading to BBB permeabilization [143]. In the SN of PD patients activated microglia presence was reported, but also in more widespread subcortical and cortical regions and were implicated in the secretion of inflammatory cytokines [144].

Furthermore, in post mortem human PD specimens and in an MPTP mouse model of PD, cytotoxic infiltration of CD8+ and CD4+ T cells and leukocytes into the CNS during the course of neuronal degeneration was described [145]. In another study, the levels of circulating Th2, Th17, and regulatory T cells were decreased, whereas T naive cells favoured the differentiation into Th1 lineage, which was accompanied by increased levels of IFN-gamma and TNF-α [146]. Remarkably, a pro-inflammatory profile of immune markers in the serum of PD patients is correlated to a faster decline in motor function and cognitive scores [147]. Moreover, in newly diagnosed PD patients systemic NLRP3 inflammasome activation and plasma ASYN levels were increased which was correlated with motor severity and progression in PD [148]. Neurotoxins, ASYN aggregation and mitochondrial dysfunction are all key regulators of NLRP3 inflammasome assembly and activation, leading to IL-1β and IL-18 release as well as pyroptotic cell death of neurons in the SN [149]. A recent study demonstrated that the stimulation of selective degradation of dysfunctional mitochondria, so-called mitophagy, in a MPTP PD model suppressed NLRP3 inflammasome activation in microglia, rescued DA neuronal loss and improved behavioural parameters [150]. Moreover, mitochondrial toxins, such as 6-Hydroxydopamine (6-OHDA), MPTP, and rotenone, trigger microglial activation accompanied by a proinflammatory cytokine production in the striatum and SN suggesting that a primary damage to the mitochondrial respiratory chain represents, alone, a trigger for neuroinflammatory processes [151,152,153].

Interestingly, the oxidative state of the CNS can be regulated by the gut microbiota via the production of various metabolites, including SCFAs; via the production of CNS neurotransmitters; and via the regulation of the permeability of the gut barrier and BBB [154]. Locally intestinal oxidative stress can promote intestinal barrier dysfunction, contributing to the translocation of bacteria and their products which trigger immune responses and inflammation thereby leading to ASYN accumulation in the ENS [105]. The higher gut permeability found in people with PD is correlated with an increase in gut proinflammatory signalling. An increased expression of TLR4 and CD3+ T cells together with the disruption of junctional complex proteins such as ZO-1 were found in colonic samples from subjects with PD [155]. Interestingly, in the same report TLR4 KO mice treated with PD inducer rotenone were protected from GI inflammation and ASYN aggregation which was translated to SN where no degeneration was observed and was associated with no motor dysfunction. Pro-inflammatory cytokines were also found to be increased in colonic biopsies from PD patients comparatively to age-matched healthy controls confirming a pro-inflammatory profile in the ENS of these patients [113]. Intestinal biopsies of PD patients also show significant differences in the intestinal mucosa and marked reductions in anti-inflammatory butyrate-producing bacteria together with the accumulation of ASYN in enteric neurons, approximately 2–5 years before developing cardinal symptoms of PD [105]. Additionally, in the stools of PD patients, an elevated level of IL-1α, IL-1β, and C-reactive protein was reported [156]. In another study, a faecal marker of intestinal inflammation, calprotectin was increased in PD patients [157]. Furthermore, in 6-OHDA exposed rats, increased oxidative stress, increase in pro-inflammatory cytokine levels, and activation of enteric glia and inflammatory cells were also observed in the colon [158]. Uncovering of these studies explains the critical link between oxidative stress and inflammation in both the GI tract and the brain of PD patients.

Furthermore, gut microbiota may produce neurotoxic metabolites, including LPS and amyloid proteins, which may reach the CNS via the systemic circulation or the vagus nerve promoting neuroinflammation and ROS production [37]. Remarkably, intranigral or systemic injection of LPS in animals selectively induces delayed and progressive loss of DA neurons in the SN [159,160]. Moreover, prenatal exposure to LPS leads to offspring with less and abnormal DA neurons [161].

#### 2.2.3. Is Microbial Infection a Risk Factor for Parkinson’s Disease?

The fact that gastrointestinal dysfunction occurs very early in PD patients, long before the clinical diagnosis is established, sheds light to the involvement of intestinal microbiome alterations in PD. Remarkably, data collected regarding the distribution of the gut microbiome in PD patients favour a high prevalence of bacteria that were correlated with PD severity, medication, and non-motor symptoms such as *Eggerthella*, *Prevotella*, *Turicibacter*, *Lactobacillus*, and *Enterococcus* [162]. Moreover, an interesting report demonstrated a positive correlation between the presence of Gram-negative *enterobacteriaceae* that were shown to secrete pro-inflammatory LPS, intestinal ASYN and increased intestinal permeability in PD [105]. On the other hand, bacteria participating in anti-inflammatory and antioxidative pathways are decreased, such as the ones from the *Prevotellaceae* family, known to secrete SCFAs [163], reduction in which leads to increased gut permeability to bacterial or microbial toxins. Interestingly, the number of *Prevotella* bacteria is negatively correlated with the severity of PD symptoms [117]. Another interesting report showed that gut levels of *Helicobater pylori* (inflammation-inducing microbes known to be involved in peptic ulcers formation) are enhanced in both acidic gastric mucosa and the basic intestinal environment of PD patients. Remarkably, *H. pylori* infection seems to influence motor function and to have a role in delaying the absorption of L-Dopa into the systemic circulation [164]. In addition, several reports observed a high prevalence of small intestinal bacterial overgrowth (known as SIBO) in PD, indicating a possible correlation with worse gastrointestinal symptoms and motor function [165,166]. Overall, these reports indicate a causative association between the microbiota–gut–brain axis and progression of the disease. Yet, the role of other microbes, such as archaeal, fungal, and viral communities in the pathogenesis of PD, has been often left largely neglected. For instance, DNA virus families such as *Herpesviridae* and RNA virus families such as *Retroviridae* and *Rhabdoviridae* were shown to be associated with parkinsonism-like symptoms [167].

Accumulating evidence have reinforced the idea that an increase in intestinal permeability can allow the translocation from the gut to the systemic circulation of microorganisms or their components such as LPS, the major outer membrane component of Gram-negative bacteria. In fact, in the colon and plasma of PD patients, the levels of LPS were found to be increased, resulting in the activation of an inflammatory response [155]. Interestingly, LPS also affects mitochondrial function by increasing mitochondrial ROS and mitochondrial fission [168]. Furthermore, LPS has been shown to disrupt the BBB and promote ASYN aggregation in the brain [169].

Similarly, metabolites derived from microorganisms can also be translocated from the gut such as b-N-methylamino-L-alanine (BMAA), a neurotoxin produced by *Cyanobacteria*. Interestingly Braak proposed a dual hit hypothesis arguing that in some cases PD pathology is likely to start in the ENS and progresses to different parts of the CNS including the SN via the vagus nerve and the olfactory bulb [170]. Pathogens (virus or bacterium) can enter the gut via the oral or nasal cavity and aggravate LB pathology in the gut resulting in the occurrence of prodromal non-motor symptoms. In fact, chronic exposure to BMAA through consumption of BMAA-contaminated foodstuffs caused amyotrophic lateral sclerosis/parkinsonism dementia complex (ALS/PDC) [171]. Moreover, BMAA has been found to be increased in the brain of patients with ALS/PDC [172]. This bacterial neurotoxin was reported to activate the metabotropic glutamate receptor 5, ultimately leading to increased oxidative stress [173,174]. Continuous intravenous injections of neurotoxic L-BMAA induced mitochondrial alterations, astrogliosis, and motor neuronal death [175]. Most importantly, a recent report from our group demonstrated that BMAA oral administration in WT mice increased intestinal inflammation, loss of intestinal barrier integrity and caudo-rostral progression of ASYN. Furthermore, we observed that BMAA induced mitochondrial dysfunction leading to neuroinflammation, dopaminergic neuronal loss, and motor deficits [176]. Considering the above, accumulating evidence suggests that some members of the gut microbiota may produce toxins targeting the mitochondria of the ENS and CNS that could result in subsequent neurodegeneration [139]. All these findings corroborate the fact that PD, in some cases, can start in the gut before it is identified in the brain, either through accumulation of bacterial metabolites and/or alterations in the gut microbiota. Moreover, these discoveries also emphasize the outstanding possibility that PD can be diagnosed early on, in the gut.

## 3. Targeting Microbiota as a Novel Therapeutic Strategy

Gut microbiota are extremely dynamic and can be modified by genetic and environmental factors, such as exercise, diet, stress, and contaminants. On the other hand, gut microbiota influence human health by regulating metabolism, host immune response, inflammatory machinery, and detoxification mechanisms [177]. Because alteration of the gut microbiota can induce changes in brain activity a new avenue for potential therapeutic manipulation of the microbiome in neurodegenerative disorders such as AD and PD has emerged. There are multiple gut microbiota interventions, including the administration of antibiotics, probiotics, prebiotics, or faecal microbiota transplantation (FMT).

### 3.1. Antibiotics

Antibiotic treatment has been reported to change disease course in both PD and AD. For instance, minocycline was shown to have neuroprotective effects in the MPTP mouse model of PD by preventing nigrostriatal DA neurodegeneration and by blocking dopamine depletion in the striatum [178]. PD patients with *Helicobacter pylori* infection showed lower levodopa absorption, antibiotic treatment reduced gastritis, improved motor fluctuations, and levodopa pharmacokinetics [179]. On the other hand, a more recent trial reported that *Helicobacter pylori* eradication does not improve clinical outcomes in PD [180]. In AD patients, the elimination of *Helicobacter pylori* by a triple eradication regimen which included 2 antibiotics (clarithromycin, and amoxicillin) and omeprazole (used to treat gastric and duodenal ulcers) improved cognitive and functional parameters [181]. A study on healthy subjects pretreated with scopolamine to mimic AD showed a positive effect of D-cycloserine at low doses [182]. Moreover, the same antibiotic improved cognitive deficits in AD patients [183]. In APP/PS1 transgenic AD mouse model, the use of a long-term broad-spectrum combinatorial antibiotic treatment reduced Aβ plaque deposition [184]. Moreover, rifampicin administration in AD mice models and in humans was shown to reduce Aβ and pro-inflammatory cytokines brain levels [185]. Likewise, minocycline, which combines anti-inflammatory and antioxidant properties, showed similar effects, but also reduced microglia activation [186]. More recently, 5 × FAD mice treated with a mixture of antibiotics displayed attenuated hippocampal Aβ pathology and decreased neuronal loss, thereby delaying memory deficits [187].

However, all these antibiotics showed controversial results in clinical trials [188,189]. In line with this, antibiotics, besides their beneficial effect in some circumstances, are also potentially harmful agents, as their overuse has been linked to microbiota impairment and related disorders. In fact, streptozotocin have been used to induce sporadic AD in animal models [190]. Moreover, several studies reported a correlation between long-term use of antibiotics and increased risk for developing PD [191,192].

### 3.2. Probiotics and Prebiotics

Probiotics, called “good” microbes are specific microorganisms that produce beneficial effects on the host health by restoring microbiota and maintaining immune homeostasis, whereas prebiotics are soluble dietary fibres that beneficially affect the host by stimulating the growth and/or activity of specific bacteria in the gut.

The supplementation of a probiotic mix for 28 days improved the intestinal permeability of AD patients [193]. A randomized, double-blind, and controlled clinical trial was conducted in AD patients to assess the effects of probiotic supplementation and observed a positive effect on cognitive function and metabolic state [194]. Another clinical trial found that probiotic and selenium co-supplementation for 12 weeks improved cognitive function and some metabolic profiles in AD patients [195]. In an open-label, single-arm study oral supplementation of *Bifidobacterium breve A1* in mild cognitive impairment participants improved cognitive function [196]. However, the supplementation in capsules containing another probiotic mix for 12 weeks did not improve memory scores, inflammatory and oxidative stress markers in AD patients [197]. In APP/PS1 transgenic mice subjected to exercise training and probiotic treatment AD progression slowed down [198]. In 3 × Tg-AD mice treated with a SLAB51 probiotic formulation showed reduction in cognitive decline due to a reduction in brain damage and reduced accumulation of Aβ aggregates [199]. In addition, D-Galactose-induced AD rats which were orally administered with *Lactobacillus plantarum* MTCC1325 not only ameliorated cognition deficits, but also decreased pathological hallmarks such as amyloid plaques and tau tangles [200]. Prebiotic administration in AD transgenic mice had similar effects alleviating AD-like symptoms by targeting the microbiota–gut–brain axis [201].

In the transgenic MitoPark PD mice daily administration with probiotics significantly reduced motor impairment and preserved tyrosine hydroxylase-positive cells in the SN [202]. Similarly, a probiotic cocktail containing a mixture consisting of *Lactobacillus* and *Bifidobacterium* protected DA neurons against MPTP and Rotenone neurotoxicity partially by increasing butyrate production [203]. Remarkably, PD patients that consumed preparations containing *Lactobacillus acidophilus* and *Bifidobacterium infantis* observed a significant reduction in abdominal pain and bloating [204]. PD patients with chronic constipation receiving milk fermented with the probiotic strain *Lactobacillus casei Shirota* significantly improve stool consistency and reduced bloating and abdominal pain [205]. In another trial in 2016 PD patients consumed fermented milk containing multiple probiotic strains and prebiotic fibre for 4 weeks, and showed an improvement in bowel movements [206]. More recently, in another study, investigators observed that probiotics (specifically belonging to the Lactobacillus and Bifidobacterium genus) significantly reduced proinflammatory cytokines and ROS production, whereas increased anti-inflammatory cytokines in peripheral blood mononuclear cells isolated from patients with PD compared to healthy controls [207]. Borzabadi and co-workers found that probiotics supplementation for 12 weeks in PD patients significantly down-regulated the gene expression of IL-1, IL-8, and TNF-α, all pro-inflammatory cytokines [208]. Probiotic supplementation improved movement and metabolic parameters in PD patients [209]. Interestingly, probiotics can promote the production of antioxidants, vitamins and bioactive molecules by microbiota which can exert beneficial effects in diseases associated with oxidative stress such as AD and PD. Interestingly, vitamin E, D3, riboflavin, and vitamin B6 have shown beneficial effects in PD patients [210]. Prebiotic fibres have been shown to modify the gut milieu improving bowel motility and reducing constipation that might be very relevant for inflammation and gastrointestinal-related symptoms in PD [211]. Hence, a few studies indicated that prebiotic fibres that generate butyrate show beneficial effects in PD animal models [212].

### 3.3. FMT

FMT consists in the introduction of a faecal suspension derived from a donor into the GI of a recipient individual. Moreover, it has been shown to be effective in the treatment of *Clostridium difficile* infection and its use was approved by the FDA [213]. Sampson and co-workers showed that faecal microbiota transplantation from PD patients to an ASYN transgenic mice model displayed worse ASYN pathology and motor deficits [214]. Another study showed that FMT from healthy mice improved motor function, increased striatal DA, and decreased neuroinflammation in a MPTP mouse model [215]. Colonization of germ-free APP transgenic mice with microbiota from conventionally-raised APP transgenic mice increased cerebral Aβ pathology, while colonization with microbiota from wild-type mice was less effective in increasing cerebral Aβ levels [83]. Accordingly, FMT treatment improved cognitive deficits and reduced Aβ deposition in the brain of APP/PS1 transgenic mice [82]. Additionally, germ-free C57BL/6N mice transplanted with faecal samples from an AD patient show significantly reduced performance on Object Location Test and Object Recognition Test when compared to mice transplanted with faecal samples from a healthy volunteer [216].

FMT in PD patients was shown to temporary improve motor symptoms and gastrointestinal function [217]. In another study, FMT via colonoscopy reduced the motor and non-motor symptoms with acceptable safety in PD patients [218]. As for AD, the only report is a case-study in which an 82-year-old AD patient showed remission of *Clostridium difficile* infection symptoms after receiving a single FMT from his 85-year-old wife and a negative stool test 2 months later. Interestingly, the cognitive score and memory retention of the patient increased 2 months after FMT [219]. An AD mouse model transplanted with healthy microbiota was reported to reduce the formation of Aβ plaques and tau pathology [220]. On the other hand, a germ-free wild-type mouse transplanted with gut microbiota from a patient with AD presented less abundant metabolites related to the nervous system and reduced cognitive function [216]. FMT may be a promising prodromal therapeutic strategy; however, it shows several challenges including risk of infection transmission and maintaining the viability and diversity of bacterial population.

### 3.4. Anti-Microbial Peptides (AMPs)

A recent opportunity with therapeutic potential for prevention and treatment of AD and PD has been highlighted with AMPs. AMPs are natural bioactive small proteins produced by all living organisms as important and indispensable components of innate immune response and are part of host defence with broad-spectrum activities from antibacterial to antifungal [221]. As opposed to antibiotics AMPs are less susceptible to antibiotic resistance [222] and can discriminate between bacteria and host cell through the differences in the cell membrane structure [223]. Interestingly, the AMP Lumbricusin (NH2-RNRRWCIDQQA) showed neuroprotective effects in a 6-OHDA induced mouse PD model, ameliorating the motor dysfunction [224]. Moreover, 2 host-defence antimicrobial peptides of α-defensins (HNP-1 and NP-3A), have been shown to prevent the aggregation and misfolding of different amyloid proteins like Aβ [225].

On the other hand, as previously discussed, in vitro and in vivo studies demonstrated that Aβ oligomers have antimicrobial activity by forming fibrils that entrap pathogens and disrupt cell membranes [14]. In addition, ASYN also exhibits antibacterial activity [226]. This is interesting and poses the possibility that in these diseases the increase in either Aβ and ASYN oligomers can be an initial protective response against these pathogens.

## 4. Perspectives for the Future

**Figure 3:**
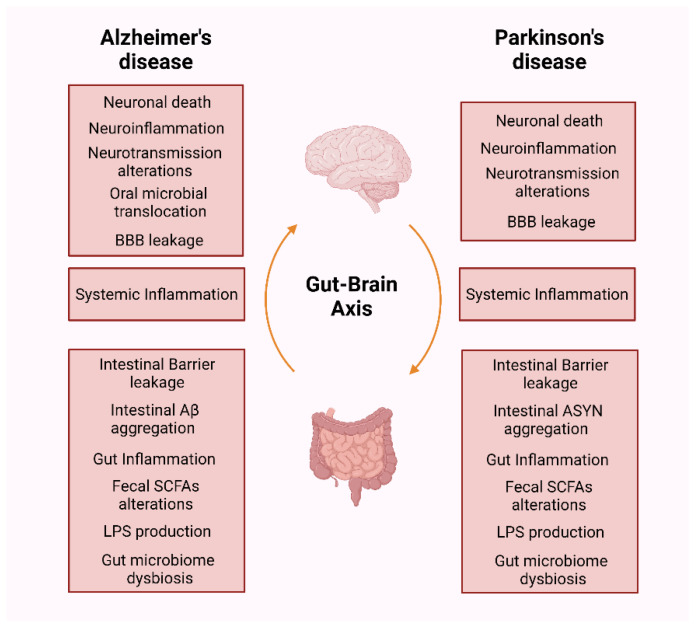
Evidence of gut–brain axis involvement in AD and PD. Schematic representation highlighting the pathophysiologic mechanisms that occur from the ENS to the CNS in AD and PD. Created by Biorender.

To date, there is no treatment designed to arrest or cure AD or PD. The present available medication does not slow or stop the progression of neurodegeneration that can develop over decades and only eases the symptoms without addressing the basic cause of the diseases. Over the years, research has extensively provided mounting evidence of the involvement of the gut–brain axis and the occurrence of neurodegenerative disorders, including AD and PD (Figure 3). Gut microbiota can indeed regulate not only the ENS but also the CNS immune response, mitochondrial function, neurotransmission, behaviour and BBB integrity. Remarkably this cause–risk effect relationship seems to be far more complex when a bidirectional route exists between the ENS and the CNS and may underline these neurodegenerative disorders progression. Furthermore, in this bidirectional route inflammation and oxidative stress are common features. In fact, as described in this review, oxidative stress and inflammation interact and cooperate to promote neurodegeneration. As a result, ongoing studies regarding gut–brain interactions in AD and PD pathological progression are vital. These studies might also bring new insight to the identification of early biomarkers before symptomatic onset occurs. In addition, the easy access to the gut and gut microbiome indicates that they can be a potential target for therapeutic intervention that arrest PD propagation into the brain. These include the manipulation of gut microbiota and microbial metabolites through probiotics and prebiotics. However, results obtained are very heterogeneous and contradictory. In fact, although generally considered safe their use may trigger side effects, infections, and allergic reactions, indicating that a stricter regulation is required towards their therapeutic usage. Interestingly, FMT may have some advantages over these supplements because its content provides a stable variety of intestinal microorganisms that can help restore the microbiome. However, in this case is also perceived a potential risk for infection and medical supervision is necessary. Overall, it is imperative to monitor and understand the exact molecular players in gut–brain axis relationship in both diseases where oxidative stress and inflammation seems to have a critical role.

## Abbreviations

Parkinson’s disease (PD); Alzheimer’s disease (AD); lipopolysaccharides (LPS); NADPH oxidase 2 (NOX2); Reactive oxygen species (ROS); Reactive nitrogen species (RNS); damage-associated molecular patterns (DAMPs); NOD-like receptors (NLRs); Toll-like receptors (TLRs); Enteric Nervous System (ENS); Central Nervous System (CNS); sporadic Alzheimer’s disease (sAD); amyloid-beta (Aβ); neurofibrillary tangles (NFT); anti-microbial peptide (AMP); pattern recognition receptors (PRR); PAMPs (pathogen-associated molecular patterns); transcription factor nuclear factor κB (NF-κB); microtubule-associated proteins (MAPs); frontotemporal lobar dementia (FTLD); Interleukin (IL); nucleotide binding oligomerisation domain, leucine-rich repeat and pyrin domain-containing protein 3 (NLRP3); blood–brain barrier (BBB); amyloid precursor protein (APP); Presenilin-1 (PS1); acetylcholine (ACh); amyloid precursor protein/presenilin-1 (APP/PS1);short-chain fatty acids (SCFAs); dynamin-1 like protein (Drp1); mitochondrial DNA (mtDNA); translocator protein (TSPO); Gastrointestinal (GI); cerebrospinal fluid (CSF); dopaminergic neurons (DA); substantia nigra (SN); alpha-synuclein (ASYN); Lewy Bodies (LBs); 1-methyl-4-phenyl-1,2,3,6-tetrahydropyridine (MPTP); 6-Hydroxydopamine (6-OHDA); small intestinal bacterial (SIBO); b-N-methylamino-L-alanine (BMAA); amyotrophic lateral sclerosis/parkinsonism dementia complex (ALS/PDC); fecal microbiota transplantation (FMT).
